# Osteogenic Potential of Human Dental Pulp Stem Cells (hDPSCs) Growing on Poly L-Lactide-Co-Caprolactone and Hyaluronic Acid (HYAFF-11^TM^) Scaffolds

**DOI:** 10.3390/ijms242316747

**Published:** 2023-11-25

**Authors:** Julia K. Bar, Anna Lis-Nawara, Tomasz Kowalczyk, Piotr G. Grelewski, Sandra Stamnitz, Hanna Gerber, Aleksandra Klimczak

**Affiliations:** 1 Department of Immunopathology and Molecular Biology, Wroclaw Medical University, Borowska 211, 50-556 Wroclaw, Poland; anna.lis-nawara@umw.edu.pl (A.L.-N.); piotr.grelewski@umw.edu.pl (P.G.G.); 2 Laboratory of Polymers and Biomaterials, Institute of Fundamental Technological Research (IPPT PAN), Polish Academy of Sciences, Adolfa Pawińskiego 5B St., 02-106 Warsaw, Poland; tkowalcz50@ippt.pan.pl; 3 Laboratory of Biology of Stem and Neoplastic Cells, Hirszfeld Institute of Immunology and Experimental Therapy Polish Academy of Sciences, R. Weigla 12, 53-114 Wroclaw, Poland; sandra.stamnitz@hirszfeld.pl; 4 Department of Maxillofacial Surgery, Wroclaw Medical University, Borowska 213, 50-556Wroclaw, Poland; hanna.gerber@umw.edu.pl

**Keywords:** dental stem cells, hDPSCs, osteogenesis, PLCL scaffold, HYAFF-11 scaffold

## Abstract

Bone tissue engineering using different scaffolds is a new therapeutic approach in regenerative medicine. This study explored the osteogenic potential of human dental pulp stem cells (hDPSCs) grown on a hydrolytically modified poly(L-lactide-co-caprolactone) (PLCL) electrospun scaffold and a non-woven hyaluronic acid (HYAFF-11™) mesh. The adhesion, immunophenotype, and osteogenic differentiation of hDPSCs seeded on PLCL and HYAFF-11™ scaffolds were analyzed. The results showed that PLCL and HYAFF-11™ scaffolds significantly supported hDPSCs adhesion; however, hDPSCs’ adhesion rate was significantly higher on PLCL than on HYAFF-11™. SEM analysis confirmed good adhesion of hDPSCs on both scaffolds before and after osteogenesis. Alizarin red S staining showed mineral deposits on both scaffolds after hDPSCs osteogenesis. The mRNA levels of *runt-related transcription factor 2 (Runx2)*, *collagen type I (Coll-I), osterix (Osx), osteocalcin (Ocn), osteopontin (Opn)*, *bone sialoprotein (Bsp)*, and *dentin sialophosphoprotein (Dspp)* gene expression and their proteins were higher in hDPSCs after osteogenic differentiation on both scaffolds compared to undifferentiated hDPSCs on PLCL and HYAFF-11™. These results showed that PLCL scaffolds provide a better environment that supports hDPSCs attachment and osteogenic differentiation than HYAFF-11™. The high mRNA of early osteogenic gene expression and mineral deposits observed after hDPSCs osteogenesis on a PLCL mat indicated its better impact on hDPSCs’ osteogenic potential than that of HYAFF-11™, and hDPSC/PLCL constructs might be considered in the future as an innovative approach to bone defect repair.

## 1. Introduction

Critical-size bone defects derived from orthopedic or oral–maxillofacial surgeries following traumas or tumor resection are very challenging pathological conditions requiring extensive bone regeneration [1,2]. The standard clinical therapy offers autograft and allograft transplantations; however, they are limited due to postoperative complications [1]. Autografts are free from immunogenic rejection and risks of disease transmission; however, their use is restricted because of limited bone resources and the risk of harvesting site morbidity leading to various complications, including infection, nerve and vascular injuries, and chronic pain [2]. Another option is a bone allograft, which is taken from a donor and can be obtained in greater quantities compared to autografts. The risk of disease transmission is negligible due to the medical protocols of harvesting, collection, and storage; however, these grafts integrate with bone defects more slowly and to a lower degree than autografts [2]. To overcome these limitations, such as deficient bone supply and donor site morbidity, it is necessary to look for alternative methods of bone regeneration. A new approach to bone repair and reconstruction, widely studied during the last three decades, is tissue engineering (TE) [3]. A tissue engineering strategy based on combined components of biomaterial/scaffolds, mesenchymal stem cells (MSCs) isolated from different tissue sources, and biologically active growth factors might create a proper microenvironment to promote cell proliferation and osteogenic differentiation [3,4].

A tissue-engineered scaffold should be biocompatible, biodegradable, and mechanically strong, and should mimic the morphological structure and chemical composition of the extracellular matrix (ECM), which accommodates the cells and creates a favorable environment by supplying nutrients, oxygen, and growth factors for new tissue formation [5]. When a scaffold is designed, the composition of the bone tissue should be considered, especially its chemical parameters, which allow for control over the physical and structural characteristics of new biomaterial to improve stem cell adhesion, proliferation, and differentiation [2]. The main mineral bone ECM component is hydroxyapatite (65–70%), whereas organic constituents of bone (30–35%) are glycoproteins, proteoglycans, and sialoproteins [2,6,7]. These molecules form a firm and crystallized structure together with collagen fibers type I, II, and V, which are dominant in ECM and play a fundamental role in the mechanical strength of bone tissue [2]. In this context, biomaterial for bone tissue engineering applied to a living organism, such as a human, must have specific features. The material needs to be biocompatible to exist in harmony with the host’s tissues and not cause harmful effects in the host [2,4]. Scaffolds should offer a favorable architecture, especially regarding porosity, pore size, and shape, and must allow for cell colonization, growth, differentiation, and vascularization [2,6]. The biological and chemical features of scaffolds should be similar to repair native tissue and allow the MSCs to release autocrine and paracrine molecules affecting the surrounding and damaged tissue as well as facilitate the proliferation and infiltration of neighboring stem cells and osteoblasts [4,6,7,8]. In this way, they can improve ECM remodeling, stimulate cell differentiation, and directly affect the rate of vascularization and bone regeneration [2,4,8]. To achieve the regeneration of the tissue and the complete substitution of exogenous material with new healthy physiological tissue, the scaffold must be biodegradable [2,9]. Moreover, the implanted biomaterials should provide enough mechanical stability at the time of implantation and not induce an immune response during scaffold degradation [6]. Finally, scaffolds should be replaced by regenerative tissue while retaining the shape and form of the regenerated tissue structure [6,9]. Many reports have discussed the usefulness of different natural and synthetic scaffolds in bone engineering and pointed out their advantages and disadvantages [2,4,10,11,12,13,14,15]. Natural polymers such as collagen, chitosan, hyaluronan, agarose, elastin, and alginate have been applied to bone tissue engineering. However, these types of scaffolds, while showing weak immune response, high flexibility, and good tensile strength, have a high risk of transmitting pathogens and mechanical instability [16,17,18,19]. Synthetic biomaterials are an alternative to natural materials for bone tissues. Different components have been used for scaffold design. Polylactic acid (PLA), polyglycolic acid (PGA), and poly e-caprolactone (PCL) are often applied in TE due to their mechanical properties, good biodegradability, and great compatibility with human MSCs [9,11,15,19]. Moreover, the synthetic materials have good reproducibility and the ability to control mechanical properties, degradation rates, and shape independently [20]. However, many synthetic biomaterials must be chemically modified to increase their adhesive properties and allow for stem cell attachment and growth [17,18]. Thus, scaffolds with multifunctional properties are required; the best option would focus on the use of hybrid systems that combine different materials [17,21]. In scaffold fabrication, different techniques have been used; however, the optimal technique should allow for the production of scaffolds with a controlled hierarchical porous structure, which has profound effects on both mechanical and biological responses of bone tissue [6]. Recently, electrospinning has attracted great attention for the fabrication of micro/nanofibrous materials similar to human tissues due to the good characteristics of the derived scaffolds [6,17]. Moreover, the physical and mechanical properties of fibrous electrospun structures can easily be modified by incorporating another polymer to develop hybrid blends or composite systems [17,22].

The intrinsic bone regeneration capacity is link with bone marrow MSC (BM-MSC) cells, which are crucial in supporting bone fracture healing through the paracrine secretion of trophic and immunomodulatory factors [1,7,23]. Studies have shown that proper osteogenesis requires not only cellular differentiation and tissue remodeling but also appropriate molecular signaling [24]. The proliferation and osteogenic differentiation of MSCs is warranted by the concurrent activity of a variety of osteogenic regulators including bone morphogenetic proteins (BMPs), fibroblast growth factors (FGFs), and transforming growth factor-β1 (TGF-β1). The osteoinductive capacity of BMPs increases the expression of osteogenic markers in MSCs at the molecular level, including early osteogenic markers such as alkaline phosphatase, runt-related transcription factor 2 (Runx2), osterix (Osx), and collagen type I (Coll-1) and late osteogenic markers such as osteopontin (Opn) and osteocalcin (Ocn) [25]. The beneficial effect of BM-MSC/scaffold application in bone regeneration has been reported in several clinical trials; however, they have some limitations, e.g., morbidity and an invasive and painful procedure during the aspiration of bone marrow for MSC isolation [9,17]. MSCs are located in specialized niches of differentiated organs and can be isolated from diverse human adult tissues (i.e., bone marrow, adipose, dental tissues) and also from perinatal tissues (umbilical cord, placenta) or fluids (amniotic liquid) [7]. A lot of reports have focused on alternative sources of MSCs such as dental-related tissues because dental stem cells (DSCs) possess many similarities to MSCs derived from bone marrow [1,15,22,26]. In heterogeneous populations of DSCs, human dental pulp stem cells (hDPSCs) show many common features with BM-MSCs, and their osteogenic properties have been intensively explored [1,19,27]. Dental tissues are identified as a rich source of mesenchymal stem/progenitor cells, which can be used for TE applications. The MSCs of dental tissue origin exhibit the minimal characterization criteria for human MSCs defined by the International Society for Cellular Therapy (ISCT) [28]. These cells express osteogenic commitment markers including Ocn, Opn, Bsp, and Dspp and can produce bone-like nodule formation in vitro [22].

In regenerative medicine, the incorporation of stem cells in scaffolds appears fundamental to the repair of many damaged tissues; however, there are only a few reports analyzing and comparing the osteogenic potential of MSCs or hDPSCs growing on different types of scaffolds including hybrid constructs [22,27,29,30,31,32]. Our recent report showed that hDPSCs seeded on a nanofibrous PLCL mat showed high osteogenic potential, as confirmed at the molecular and protein levels [22]. Despite numerous published results, there are not sufficient data to select the optimal scaffold to be incorporated with MSCs as a bioconstruct for bone engineering [8,11,14,17,20,30]. In this context, we focused on in vitro studies evaluating the osteogenic potential of hDPSCs growing on the commercially available hyaluronan acid membrane, HYAFF-11^TM^, and a poly(-L-lactide-co-caprolactone) (PLCL) scaffold.

## 2. Results

### 2.1. Adhesion of hDPSCs Grown on the PLCL and HYAFF-11™ Scaffolds

**Figure 1:**
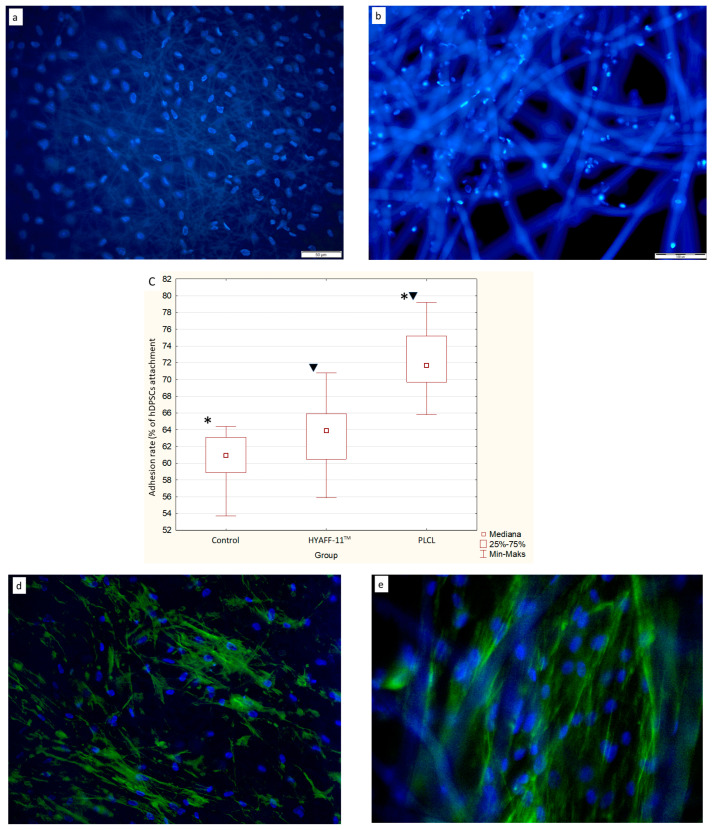
Adhesion of hDPSCs grown on PLCL and HYAFF-11™ scaffolds and as a monolayer (control). (**a**) Adhesion and distribution of hDPSCS growing for 24 h on PLCL and (**b**) HYAFF-11™, with adherent hDPSCs seeded after 24 h of cultivation. Nuclei counterstained with DAPI are shown in blue. (**c**) Adhesion rate of hDPSCs cultured on the PLCL nanofibrous membrane was significantly higher compared to the control group at 24 h (* *p* < 0.0001) as well as between PLCL and non-woven HYAFF-11™ scaffolds (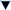 *p* < 0.001) (statistical analysis: Kruskal–Wallis test). Columns show median adhesion of hDPSCs ± SD. Three repeated assays were performed for each case (*n* = 3) and data are presented as median ± SD. (**d**) Immunofluorescence staining of hDPSCs grown on PLCL and (**e**) HYAFF-11^TM^ scaffolds by using Alexa Fluor 488 conjugated phalloidin (green) and nuclei counterstained with DAPI (blue). Scale bar = 50 µm for (**a**,**d**) and 100 µm for (**b**,**e**).

The adhesion of the hDPSCs (*n* = 3) seeded on the PLCL and HYAFF-11™ scaffolds was assessed 24 h after seeding. The median adhesion of hDPSCs seeded and grown for 24 h on PLCL scaffolds was 72.4% ± 3.67%, whereas for cells growing on HYAFF- 11™ membranes, it was 63.6% ± 3.69%, and in a 2D cultivation serving as a control group, it was 60.7% ± 2.83%. After 24 h of hDPSC cultivation, the seeded stem cells were regularly distributed on the surface of the PLCL scaffolds, as shown in Figure 1a, whereas on the HYAFF-11™ scaffolds, hDPSCs were visible on fibers and covered the fibers in different densities (Figure 1b). The adhesion rate of the hDPSCs was significantly higher on PLCL compared to the control group (*p* < 0.0001) and significant differences were also observed between PLCL and HYAFF-11™ scaffolds (*p* < 0.001) (Figure 1c). Cell adhesion was confirmed by phalloidin expression on cells attached to the respective scaffolds, identifying cytoskeleton organization by F-actin staining (Figure 1d,e).

#### Scanning Electron Microscope (SEM)

**Figure 2:**
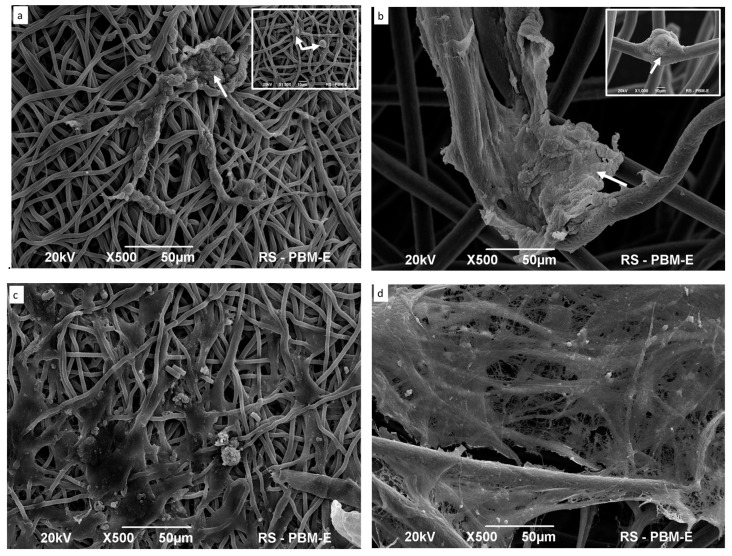
SEM images of scaffolds with seeded hDPSCs before (5 days cultured) and after (21 days cultured) osteogenic differentiation. (**a**) PLCL undifferentiated hDPSCs colonized fibers of PLCL (double arrow) and formed groups (single arrow). (**b**) Cells adhered to and covered fibers of HYAFF-11™ scaffolds. Arrow insert in (**b**) indicates cells located in the crossing of fibers. (**c**) hDPSCs differentiated on PLCL formed calcium bone matrix deposition. (**d**) hDPSCs-HYAFF-11™ construct after osteogenic differentiation and secretion of ECM and minerals, with depositions of large mineral clusters.

SEM images showed the adhesion of hDPSCs at the surface of the PLCL and HYAFF-11™ membranes without differentiation after five days of cultivation (Figure 2a,b). Both membrane fibers were covered by adherent stem cells that were easily detectable on both scaffolds. Cells encircling the PLCL and HYAFF-11™ fibers were elongated, showed a flat shape, and built the network between the fibers (Figure 2a,b). Moreover, the hDPSCs observed on PLCL formed a dense cell sheet, which grew through the pores within scaffolds (Figure 2a). The hDPSCs grown on HYAFF-11™ colonized the crossing fibers or opsonized a single fiber (Figure 2b). After 21 days of differentiation, cells intensively penetrated into the pores of both scaffolds, secreted high amounts of extracellular matrix covered membrane surfaces, and enhanced cell-to-cell interaction, and this phenomenon was observed on both scaffolds but was more clearly visible on HYAFF-11™ surfaces than on PLCL membranes (Figure 2c,d).

### 2.2. Osteogenic Differentiation of hDPSCs on PLCL and HYAFF-11™ Scaffolds

#### 2.2.1. Alizarin Red S Staining

**Figure 3:**
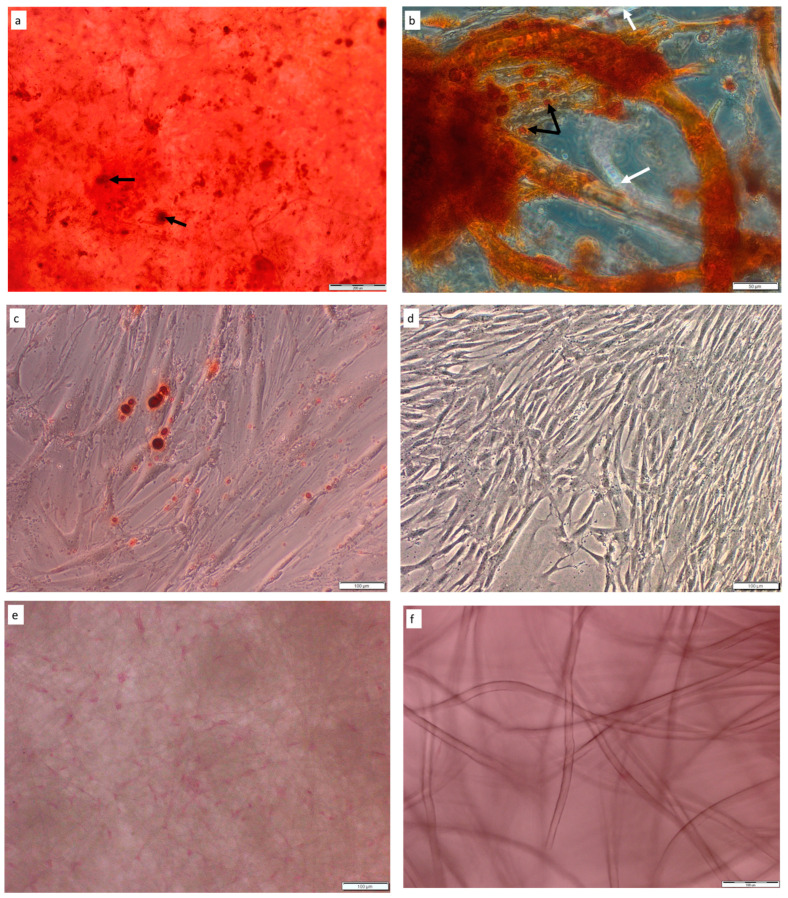
Alizarin red S staining of hDPSCs on PLCL and HYAFF-11™ scaffolds after osteogenic differentiation. (**a**) hDPSCs were covered and embedded in mineralized matrices, and mineralization nodules and mineral deposits on PLCL surfaces were visible (black arrows). (**b**) hDPSCs after osteogenic differentiation on HYAFF11™ scaffolds showed high mineral deposition, confirming the presence of matured osteoblasts (black arrows indicate the minerals secreted by hDPSCs, while white arrows refer to fibers with cells negative for alizarin red S staining. Control groups: undifferentiated hDPSCs grown in 2D culture (**d**) and on PLCL (**e**) and HYAFF-11^TM^ (**f**) scaffolds. Scale bar = 200 µm (**a**), 100 µm (**c**–**f**), and 50 µm (**b**).

Visualizations of the mineralized matrix depositions within the PLCL and HYAFF-11™ scaffolds were assessed by alizarin red S staining at day 21 after the osteogenic differentiation of hDPSCs on PLCL and HYAFF-11^TM^ and in monolayer growth. Differentiated cells had formed mineral deposits when cultured on PLCL and HYAFF-11™ scaffolds (Figure 3a,b), as well as in the 2D culture (Figure 3c). As presented in Figure 3a, differentiated hDPSCs grown on PLCL nanofiber mats formed cell groups producing mineralized matrices and showed mineralization effects distributed on the whole surface of the scaffolds, whereas, on hyaluronan scaffolds (HYAFF-11™), the mineral deposition formed clusters (Figure 3b). Moreover, calcium deposits detected by alizarin red S staining were observed on fibers of hDPSCs-PLCL and hDPSCs-HYAFF-11™ constructs, but fibers with or without calcium depositions were more visible on HYAFF-11™ scaffolds than on PLCL mats (Figure 3b). hDPSCs differentiated in 2D conditions towards osteoblasts showed mineral deposits (Figure 3c). Undifferentiated hDPSCs grown as 2D and on PLCL and HYAFF-11^TM^ scaffolds did not show alizarin red S staining (Figure 3d–f).

#### 2.2.2. Bone-Related Protein Expression

**Figure 4:**
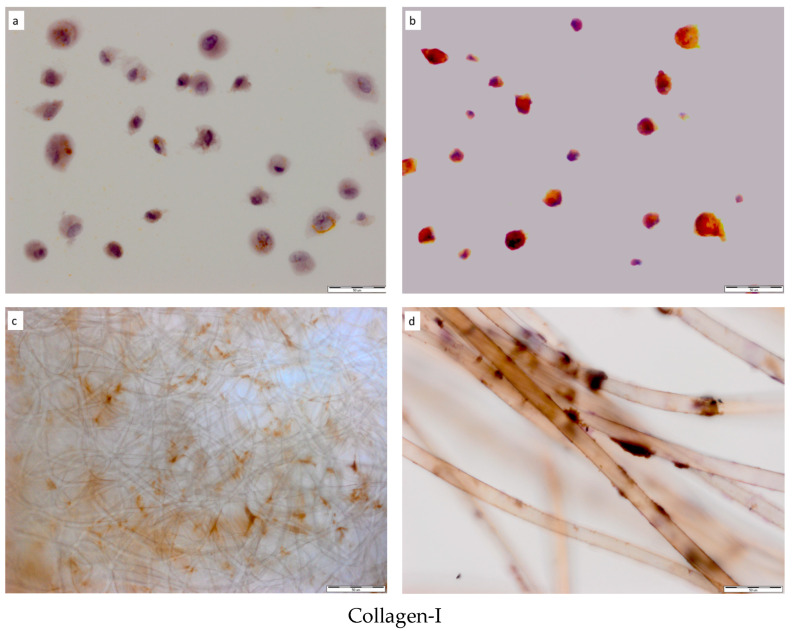

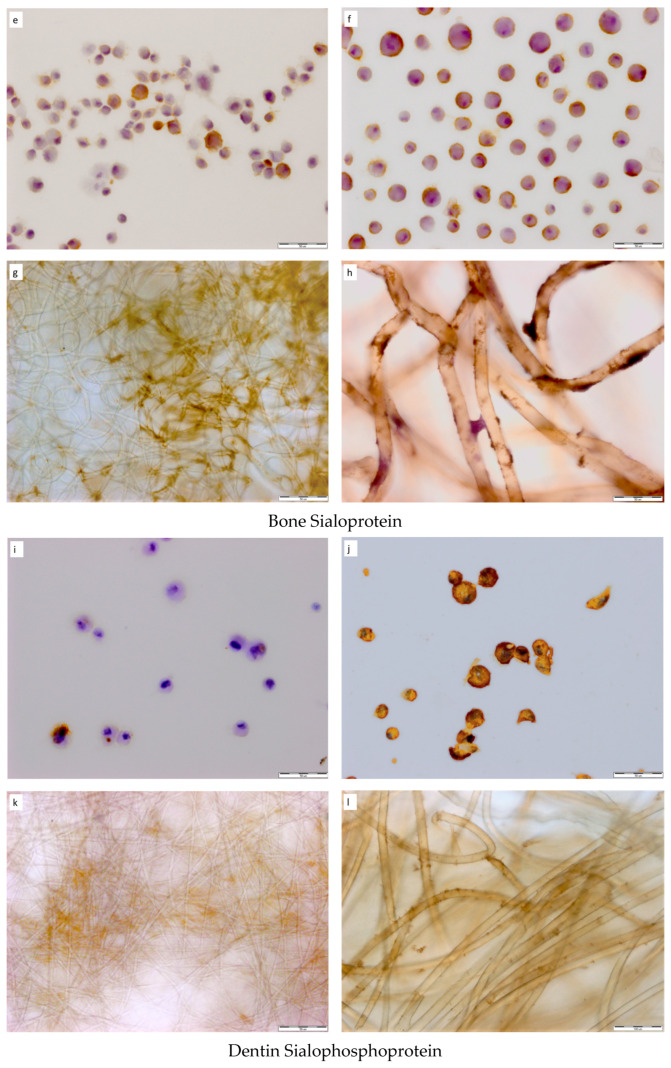

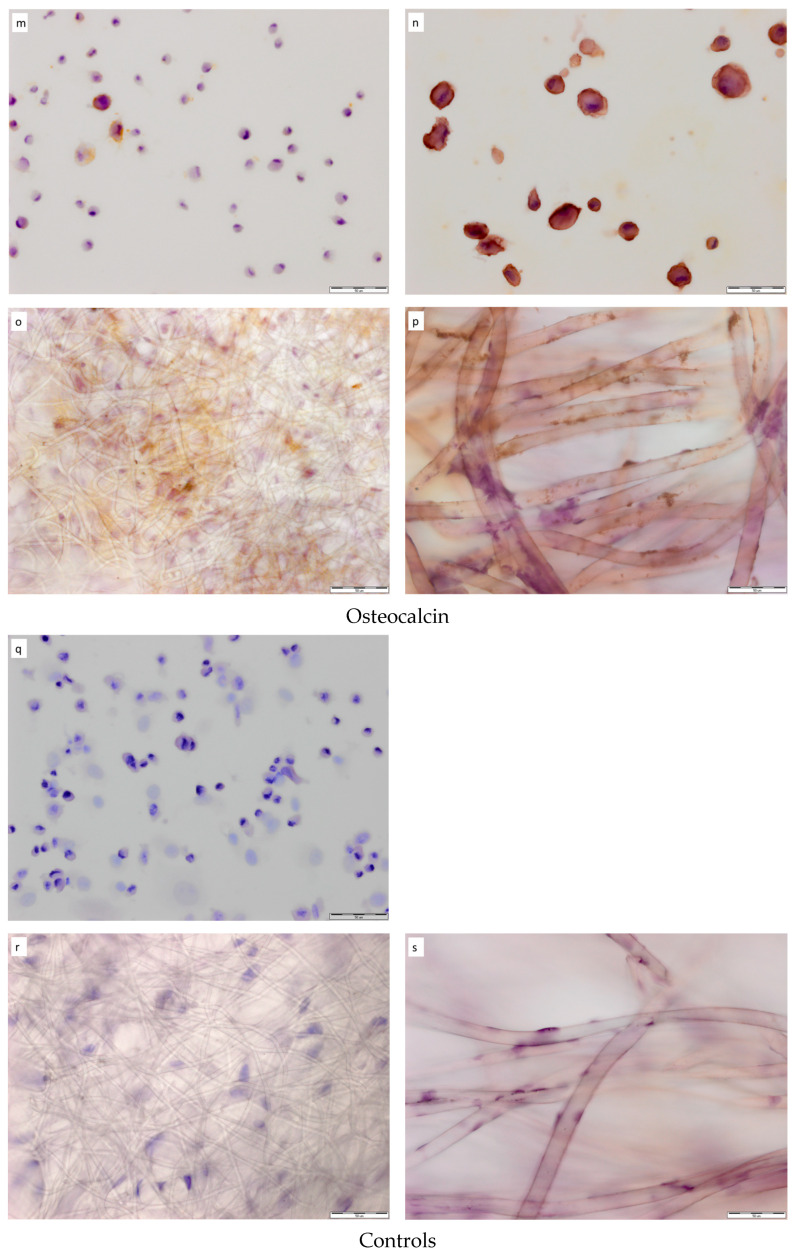
Comparison of bone-related proteins expressed by hDPSCs grown on PLCL nonfibrous and HYAFF-11™ scaffolds before and after osteogenic differentiation. (**a**) Low collagen type 1 expression in hDPSCs cytospin specimens before and (**b**) after hDPSC differentiation. (**c**) High expression of collagen type I in cells differentiated on PLCL. (**d**) The cells located on HYAFF-11^TM^ scaffold fibers showed different intensities of collagen type I immunostaining. (**e**) Cytospin specimens of undifferentiated hDPSCs with low BSP expression and (**f**) differentiated hDPSCs showing strong immunopositivity for BSP staining. (**g**) High BSP immunopositivity of cells differentiated on PLCL and (**h**) HYAFF-11^TM^. (**i**) Cytospin specimens of undifferentiated hDPSCs with low DSPP expression and (**j**) differentiated hDPSCs with high DSPP expression. (**k**) DSPP expression in hDPSCs differentiated on PLCL scaffolds and (**l**) located on fibers of HYAFF-11™ scaffolds. (**m**) Cytospin specimens of undifferentiated hDPSCs with weak expression of OCN and (**n**) differentiated hDPSCs with moderate OCN expression. (**o**) OCN expression in differentiated hDPSCs on PLCL and (**p**) on HYAFF-11^TM^. (**q**–**s**) Controls: (**q**) cytospin specimens, (**r**) hDPSC/PLCL constructs, and (**s**) hDPSC/HYAFF-11^TM^ constructs. Scale bar = 100 µm (**l**) and 50 µm (**a**–**k**,**m**–**s**).

The expression of collagen type I, BSP, OCN, OPN, OSX, DSPP, and Runx2 were analyzed on hDPSCs grown on PLCL and HYAFF-11^TM^ scaffolds before and after osteogenic differentiation. As presented in Table 1, the immunohistochemical analysis showed statistically significant differences between the collagen type I, BSP, OCN, OPN, OSX, DSPP, and Runx2 protein expression of hDPSCs grown as a 2D culture or on PLCL or HYAFF-11™ scaffolds before and after hDPSC osteogenic differentiation. Before osteogenic differentiation, hDPSCs grown on scaffolds or as a 2D culture showed weaker expression of bone-related proteins, resulting in a low proportion of positive cells for analyzed proteins than differentiated cells (Table 1). The differentiated hDPSCs on PLCL and HYAFF-11™ scaffolds expressed all analyzed proteins. The expression of all biomarkers showed heterogeneous patterns of immunostaining on both scaffolds, and collagen type I expression was significantly higher on PLCL than on HYAFF-11^TM^ (*p* = 0.0002). Such a trend was not found for the remaining proteins. Among the analyzed bone-related proteins, after osteogenic differentiation, the cells located on hDPSCs/PLCL and hDPSCs/HYAFF-11^TM^ constructs showed different ranges of immunostaining and intensities of immunoenzymatic reaction. (Figure 4a–p). As visible on the set of selected microphotographs showing early and late bone-related proteins expressed by hDPSCs after osteogenic differentiation, hDPSCs revealed high expressions of osteoblast biomarkers such as BSP, DSPP, and OCN (Figure 4e–h, i–l, and m–p, respectively) compared to undifferentiated hDPSCs (Figure 4q–s).

#### 2.2.3. Bone-Related Gene Expression

**Figure 5:**
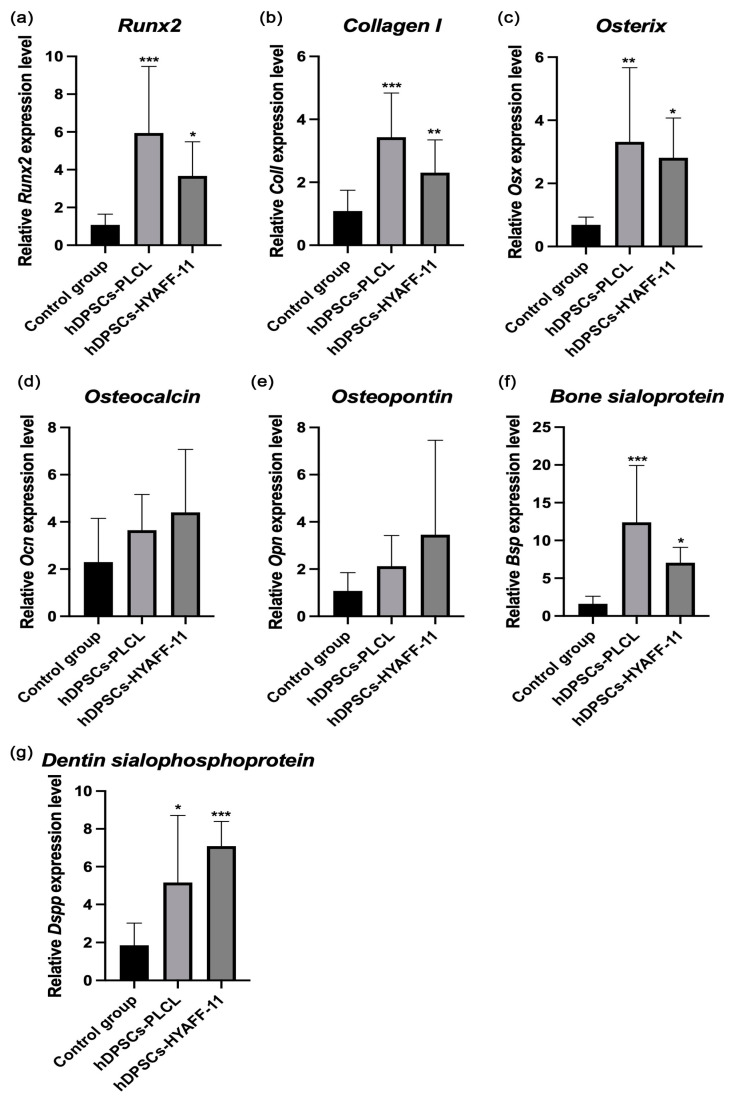
Real-time PCR analysis for the osteogenic differentiation gene markers of non-treated hDPSCs (control group) and osteogenic-induced constructs of hDPSC-PLCL and hDPSC-HYAFF-11^TM^. Relative expression levels of (**a**) *runt-related transcription factor 2* (*Runx2*), (**b**) *collagen type I* (*Coll-I*), (**c**) *osterix* (*Osx*), (**d**) *osteocalcin* (*Ocn*), (**e**) *osteopontin* (*Opn*), (**f**) *bone sialoprotein* (*Bsp*), and (**g**) *dentin sialophosphoprotein* (*Dspp*) were detected. Three different experiments were performed. * *p* < 0.05, ** *p* < 0.005, *** *p* < 0.001.

The relative gene expression levels of osteogenic markers *Runx2*, *Coll-I*, *Osx*, *Osn*, *Opn*, *Bsp*, and *Dspp* were detected in three patient samples of untreated hDPSCs and osteogenic-induced constructs of hDPSC-PLCL and hDPSC- HYAFF-11™ at day 21 of differentiation (Figure 5a–g). The results are presented as the mean value of three patient samples. *Runx2* expression levels were higher in hDPSC-PLCL constructs than in constructs of hDPSC-HYAFF-11™ (RQ 5.95 vs. 3.67, *p* < 0.005). However, both hDPSC-HYAFF-11™ and hDPSC-PLCL constructs had a higher mRNA expression of *Runx2* than the control group (RQ 1.08). Similarly, *Coll-I* expression levels were higher in hDPSC-PLCL constructs than in hDPSC-HYAFF-11™ constructs (RQ 3.43 vs. 2.30, *p* < 0.001). The highest *Osx* expression was observed in hDPSC-PLCL constructs (RQ 3.32 vs. 2.81 in hDPSC-HYAFF-11™ and 0.69 in control group, *p* < 0.005). However, hDPSC-HYAFF-11™ constructs exhibited a higher relative expression level for late osteogenic differentiation markers *Ocn* and *Opn* than hDPSC-PLCL constructs (RQ 4.40 vs. 3.65 for *Ocn* and 3.46 vs. 2.12 for *Opn,* respectively). *Bsp* expression in hDPSC-PLCL constructs was higher than in hDPSC-HYAFF-11™ constructs (RQ 12.41 vs. 7.05, *p* < 0.001). Nonetheless, in hDPSC-PLCL constructs, *Dspp* expression levels were lower than in hDPSC-HYAFF-11™ constructs (RQ 5.16 vs. 7.09, *p* < 0.05). 

## 3. Discussion

The present in vitro study was carried out to assess the osteogenic capacity of human dental MSCs growing on PLCL nanofiber and commercial hyaluronan acid (HYAFF-11™) scaffolds [12,22]. The scaffolds used in this experimental study were selected based on their physical and chemical properties, which are important for cell–scaffold communication, cell-to-cell interactions, and the activation of cell signaling pathways for cell proliferation and differentiation and the distribution of ECM proteins [9,14,33,34,35]. Dental pulp stem cells under well-defined osteogenic conditions hold the potential to differentiate into bone cells, but their osteogenic capacity depends on many factors; however, in bone engineering, it mainly depends on the scaffold architecture and chemical composition [2,4,7,14,22,36]. Several reports have documented the osteogenic capacity of hDPSCs seeded on different biomaterials, but, sometimes, hDPSCs’ differentiation towards osteoblasts is insufficient, and designed bioimplants do not support bone regeneration [14,33,36,37,38]. The results from the current study regarding hDPSC adhesion and distribution on PLCL and HYAFF-11™ scaffolds before differentiation, similarly to previous data, showed good growth and distribution of cells on PLCL and HYAFF-11™ fibers [12,22,36,39]. This finding indicates that interactions between hDPSCs and PLCL and HYAFF-11™ fibers promote hDPSC proliferation and adhesion [12,22,36]. However, as observed in the current study, a higher adhesion of hDPSCs on PLCL than on HYAFF-11™ scaffolds suggests that hDPSCs’ behavior might be related to the intrinsic characteristics of the specific mechanical and chemical parameters of the analyzed scaffolds [12,29,38]. To explain the differences in hDPSC adhesion on both scaffolds, it is worth underlining the fact that the surface morphology, e.g., porosity and fiber size of electrospun nanofibrous and chemical composites of PLCL scaffolds, is different than that of HYAFF-11™ [12,22]. Several data showed that hydrophilic surfaces showed better cellular adhesion than hydrophobic surfaces because hydrophilic surfaces support the diffusion of bioactive molecules and cellular waste from nanofibers [17,18,22,29]. Thus, we can assume that the hydrolytically modified nanofiber PLCL mats for hDPSC osteogenic differentiation used in our study might influence on cellular function for the most suitable stimulation of stem cell growth [22,34,35]. Our results revealed that the structural or chemical variation of the nanofiber PLCL and non-woven HYAFF 11™ meshes have an impact on hDPSC adhesion and growth. These results are in line with those of Alipour et al. [36], who found a better adhesion and proliferation of hDPSCs growing on poly-(ε-caprolactone)—poly (ethylene glycol-poly-(ε-caprolactone) (PCL-PEG-PCL) modified by zeolite than those growing on non-modified PLA and PLA/HA woven scaffolds. Also, Wang et al. [29] reported that hybrid nanofibrous PLCL scaffolds modified by silk fibroin facilitate human adipose-derived stem cells (hADSCs)’ ability concerning adhesion, proliferation, and osteogenic differentiation. Similar results have been presented by other groups showing that electrospun scaffolds consisting of polycaprolactone (PCL) and hydroxyapatite (HA) support the adhesion and proliferation of hADSCs [40]. Authors have indicated that PCL/HA composites enhance cell–cell communication and increase hADSC attachment to scaffolds [40].

Many reports analyzing MSC adhesion capacity on different types of scaffolds have revealed that cell adhesion and proliferation are dependent on the structural properties of the scaffold, such as pore structure and topography [12,14,33,34,35,36,37,38,41]. Our studies using SEM analysis revealed a better adhesion of hDPSCs to PLCL than to HYAFF11™ surfaces, and this phenomenon might be dependent on fiber size and the ability for cell–cell contact. It was observed that thinner fibers, characteristic of PLCL scaffolds, can provide a suitable 3D space for stem cell growth, adhesion, communication, and nutrient transportation [36]. The structural properties and composition of the scaffold play a crucial role in the biocompatibility of the cell–scaffold construct. Several studies have found that PCL, PLA, and PLCL scaffolds support stem cell growth and osteogenic differentiation [9,14,22,35,36,38,40,41], whereas hyaluronic acid-based polymers mainly support the chondrogenesis of stem cells; however, this composite also induces MSC osteogenesis [12,42]. Some authors suggest that scaffolds composed of nanofibers are structurally similar to natural bone ECMs, and this membrane might provide a better environment for stem cell proliferation and osteogenic differentiation [29,36,43]. Based on this observation and our results, we might conclude that PLCL is a better platform for hDPSC growth than HYAFF-11™ [22,36]. On the other hand, in other published reports, it has been found that hDPSC proliferation, adhesion, and osteogenic capacity are associated with MSC features derived from individual donors [22,29,38,40], and this observation is also reflected in our study. This observation was confirmed by SEM images showing hDPSC distribution on the scaffolds’ surfaces before osteogenic differentiation. According to several studies indicating that the pore size is responsible for cell infiltration inside the scaffold, presented data also found that hDPSCs migrate into the deep region of the membrane [9,12,36,38,40]. The ability of hDPSCs to differentiate into osteoblasts is one of the important features in bone engineering as mature osteoblasts are capable of depositing minerals [35,36,37,38]. Many studies have documented that after cellular maturation, matrix mineralization can be visualized by, for example, alizarin red staining and SEM analysis [34,35,36,44]. Our results revealed mineralized nodules on both scaffolds, thus confirming the osteogenic differentiation of hDPSCs on the surface of scaffolds. These observations are in line with previous reports showing that biodegradable materials, such as PLCL and HYAFF-11™ developed as scaffolds for stem cells, can generate microenvironments suitable for hDPSCs’ differentiation into osteoblasts [12,29,36]. Similarly to published data, both bioimplants induced the secretion of ECM minerals, with depositions of mineral clusters in different ranges on scaffold surfaces. The cells organized themselves in agglomerates, forming regions containing a layer of cells on the matrix surface, filling the pores and covering fibers [33,36,38,41].

To investigate the effects of PLCL nanofiber and HYAFF-11^TM^ scaffolds on the osteogenic differentiation of hDPSCs and subsequent creation of the alizarin red-positive mineral deposits that appeared on analyzed scaffolds, in this study, the expression levels of osteogenic genes involved in hDPSCs’ differentiation into osteoblasts such as *Runx2, Coll-I*, *Osx*, *Ocn, Opn, Dspp*, and *Bsp* were assessed using qRT-PCR. Our data showed that PLCL scaffolds exert superior effects on hDPSC osteogenic differentiation compared to HYAFF-11™. The hDPSC-PLCL constructs appeared to have significantly higher expression of the early (*Runx2, Coll-I*, *Osx*) and late (*Opn*, *Bsp*) osteogenic markers than hDPSCs differentiated on HYAFF-11™ at the end stage of osteogenic differentiation at day 21. Similar observations were reported in studies on the osteogenic differentiation of different tissue-derived MSCs on PCL nanofiber scaffolds and revealed increased osteogenic gene expression, including for *Runx2* and *Coll-I*, at three weeks after osteogenic differentiation [45]. The high *Runx2* expression found in our study after 21 days of osteogenic differentiation of hDPSCs is in line with Alipour et al.’s studies [33] showing increased expression of *Runx2* at day 21, which was maintained after 28 days of hDPSC osteogenic differentiation. This significant *Runx2* upregulation was observed in the Alg/Gel/nHA microcapsules and was 5.1-fold higher compared to the control group. Based on our’ and others’ results, we might assume that a three-dimensional microenvironment created by hydrolytically modified PLCL could upregulate osteogenic gene expression in hDPSCs. The activity of bone–related gene expression has been confirmed immunohistochemically for corresponding protein expression. The expression of the specific proteins encoded by analyzed genes was observed in differentiated cells found on both scaffolds. The osteogenic-related gene expression and their related protein expression found in this study point out that DPSCs grown on PLCL and HYAFF-11™ scaffolds possess the capacity to differentiate into cells that express osteoblast phenotypes, and showed a high mineralization capacity, as confirmed by osteocalcin expression, defined as a marker of mineralization [34,40,41]. In our study, osteogenic gene expression markers were represented at different levels after 21 days of hDPSC osteogenic differentiation on PLCL and HYAFF-11™ scaffolds. These results are in agreement with published reports that suggest that the expression of osteoblast markers depends on the time of osteoblast maturation and that indicate that *Runx2, Bsp*, and *Osx* are expressed in the early stage of osteoblast differentiation (by committed osteoprogenitors and early osteoblasts) and are important in the stabilization of the bone matrix structure; however, *Coll-I* is also expressed by osteoprogenitors and early osteoblasts during the initial period of proliferation and ECM-synthesis [26]. This observation might be supported by the high *Coll-I* expression at the gene and protein levels found in the current study.

In turn, *Ocn* and *Opn*, as late indicators of osteoblast development that play key roles in the onset of matrix mineralization, expressed by hDPSCs after differentiation in our study, indicated that hDPSCs change phenotypes into osteoblasts and might secrete different bone products that can act as a pattern of mineral deposition [35,40,44].

We can assume that hydrolytically modified PLCL nonfibrous and non-woven hyaluronic acid HYAFF-11™ scaffolds provided a suitable microenvironment for the intracellular signaling activity in the cell–cell and cell–fibers of each scaffold, inducing the extracellular matrix synthesis and osteogenic potential of hDPSCs and the mineralization of ECM, and similar observations have been reported by others [12,35,36,38].

## 4. Materials and Methods

### 4.1. Biomaterials

#### 4.1.1. Hyaluronan-Based Biomaterial (HYAFF-11™)

In the present study, a commercially available non-woven mesh of hyaluronan benzyl ester derived from the total esterification of sodium hyaluronate (synthesized from 80 to 200 kDa) with benzyl alcohol and named HYAFF-11™ (Fidia Advanced Biomaterials, Abano Terme, Padova, Italy) was used. The HYAFF-11™ fibers were fabricated by extraction and had a diameter of about 50 µm and a specific weight of 100 g/m^2^. The properties of these substrates are described in detail elsewhere [46].

#### 4.1.2. Poly(L-Lactide-Co-Caprolactone) (PLCL) Scaffold

The material used in this study was a PLCL nanofiber mat (Purasorb 7015, Purac-Corbion, Amsterdam, The Netherlands) composed of 70% L-lactide and 30% caprolactone units with an inherent viscosity of 1.5 dl/g that is GMP certified and used for the production of medical devices. Electrospinning was carried out in a Fluidnatek LE-50 chamber equipped with a humidity and temperature control module (Bioinicia, Valencia, Spain), as described earlier [22]. The PLCL nanofiber mat was hydrolytically modified by dipping it in a 10% NaHCO_3_ aqueous solution for four days at 37 °C.

### 4.2. Stem Cell Study-Related Methods

#### 4.2.1. Patients

From three healthy individuals (aged 15–22 years), the third molars were routine extracted and human dental pulp was aspirated. All participants of the study provided informed signed consent following a detailed explanation of the research protocols. All procedures regarding pulp tissue collection and the in vitro study of dental pulp stem cells were approved by the Ethics Committee of the Medical University in Wroclaw, Poland (no. KB-127/2023).

#### 4.2.2. hDPSC Isolation and Culture

Dental pulp tissue was gently removed from the teeth and immersed for 1 h at 37 °C in a digestive solution containing 3 mg/mL of collagenase type I from Clostridium histolyticum (Sigma-Aldrich, St. Louis, MO, USA) and 4 mg/mL of Dispase II (Gibco, Life Technologies, New York, NY, USA) [13,21]. hDPSCs were cultured in culture flasks/dishes at 37 °C and 5% CO_2_ in the α-minimal essential medium (α-MEM; Gibco, Karlsruhe, Germany) supplemented with 20% fetal calf serum (FBS) (Gibco, Karlsruhe, Germany), 100 IU/mL penicillin, and 100 μg/mL streptomycin (Sigma-Aldrich, St. Louis, MO, USA), as described in our previous study [22]. The MSC characteristics of the hDPSCs were confirmed according to the criteria of the International Society for Cellular Therapy [6]. The three main features of the hDPSCs were as follows: adherence to plastic, expression of specific cluster differentiation markers (CD), and trilineage differentiation potential for chondrogenesis, osteogenesis, and adipogenesis, confirmed by our earlier investigations [22]. All experiments were performed using hDPSCs at the third passage in two replicates.

### 4.3. hDPSC Seeding on PLCL and HYAFF-11™ Scaffolds

hDPSCs were seeded on the PLCL and HYAFF-11™ membranes to create stem cell–scaffold constructs by using our well-established method, as previously described [12,22]. Briefly, hDPSCs at the density of 5 × 10^4^ were resuspended in 50 μL of a culture medium and carefully dripped onto precut PLCL 0.5 × 0.5 cm PLCL and HYAFF-11™ scaffolds, placed in Petri dishes, and incubated for 2 h at 37 °C. Next, 2 mL of the α-MEM medium was added and cultured for seven days. The medium was changed twice a week. Both cell–scaffold constructs were analyzed under an inverted microscope to assess the cells’ adhesion to the scaffold surface. hDPSCs from all donors (*n* = 3) were used to prepare the hDPSC-PLCL and hDPSC-HYAFF-11™ constructs.

#### 4.3.1. Adhesion of hDPSCs to the PLCL and HYAFF-11™ Scaffolds

hDPSCs were seeded onto the PLCL and HYAFF-11™ scaffolds at a density of 1 × 10^3^/cm^2^ in six-well plates and cultured in the α-MEM medium at 37°C with 5% CO_2_ for 24 h. hDPSCs cultured at the same time point without a scaffold served as a control. The adherent hDPSCs were enzymatically (trypsin 0.25%-EDTA) removed from the PLCL and HYAFF-11™ scaffolds and counted using an Automated Cell Counter mark R1 (Olympus, Tokyo, Japan). The hDPSCs’ adhesion rate was shown as a percentage of the initial number of cells seeded onto scaffolds. Next, the cells were stained with 4’,6-diamidino-2-phenylindole (DAPI). Finally, the hDPSCs’ adhesion while growing on the scaffolds was observed using a fluorescence microscope (BX61, Olympus, Japan). The experiments were repeated three times in three replicates for each hDPSC case (*n* = 3). Additionally, the cytoskeletons of hDPSCs grown on PLCL and HYAFF-11^TM^ scaffolds were determined by F-actin staining before differentiation. hDPSCs on PLCL and HYAFF-11^MT^ scaffolds were fixed for 15 min at RT in a 10% formalin solution (Merck, Saint Louis, MO, USA). After that, the cells on both scaffolds were treated with 0.1% Triton™ X-100 for 15 min and washed with PBS three times. hDPSCs were incubated with Alexa Fluor 488-phalloidin (Thermo Fisher Scientific, Rockford, IL, USA) (dilution of 5 µL of the methanol stock solution in 200 µL of PBS) for 40 min. Next, cell nuclei were stained with DAPI. Finally, the hDPSCs’ adhesion onto PLCL and HYAFF-11^TM^ scaffolds was assessed using a fluorescence microscope (BX61, Olympus, Japan).

#### 4.3.2. Scanning Electron Microscopy Analysis

The materials used in the experiments were fixed in a sequence of solutions: first, in 2% paraformaldehyde, followed by 2.5% glutaraldehyde in a cacodylate buffer and then in 1% osmium tetroxide with potassium ferricyanide for 2 h. Next, the materials were post-fixed in a 1% OsO_4_ solution, dehydrated with a series of ethanol solutions, and dried using liquid CO_2_ in a critical point dryer. Finally, the material was coated with gold, and images were captured using a JSM-6390LV scanning electron microscope manufactured by Jeol in Tokyo, Japan.

#### 4.3.3. Osteogenic Potential of hDPSCs Grown on PLCL and HYAFF-11™ Scaffolds

For the analysis of osteogenic potential, hDPSCs from three donors were seeded 5 × 10^4^ cells/cm^2^ on PLCL and HYAFF-11™, maintained in a complete hMSC osteogenesis induction medium, FCS-kit (Provitro, Germany), and cultured at 37 °C with CO_2_ for 21 days, according to the manufacturer’s protocol as follows: 50 mL of osteogenic induction basal medium was prepared by adding 5 mL FCS, 1 mL HEPES, 0,5 mL L-glutamine, and 350 µL penicillin and streptomycin. Next, it was supplemented by osteogenic induction factors: 500 µL dexamethasone, 500 µL β-glycerol–phosphate, and 500 µL ascorbic–acid-2-phosphate. The process of hDPSC differentiation was carried out for 21 days at 37 °C in a 5% CO_2_ atmosphere. hDPSCs differentiated in a 2D culture and undifferentiated hDPSCs on PLCL and HYAFF-11^TM^ served as controls. The induction medium was changed twice a week.

### 4.4. Alizarin Red S Staining

After 21 days of hDPSC differentiation on PLCL and HYAF-11™ and 2D cultured dishes, the functional status was confirmed by alizarin red S (Sigma-Aldrich, St. Louis, MO, USA) staining to show the rate of mineralization and detect the mineral deposition. After 21 days of cultivation, the constructs of cells/PLCL, cells/HYAFF-11™, and 2D cultured dishes were washed with PBS, fixed in a 10% formalin solution, washed in PBS again, and incubated with alizarin red S for 30 min in a dark chamber at RT [22]. Undifferentiated hDPSCs growing on PLCL and HYAFF-11^TM^ and in a 2D culture were negative controls. Finally, the bioimplants and 2D dishes with the differentiated and undifferentiated cells were washed in PBS and analyzed using an Olympus IX73 microscope (Olympus, Tokyo, Japan).

#### 4.4.1. Antibodies

For immunohistochemical staining, the following antibodies were used: anti-osteocalcin (OCN) (mouse monoclonal, IgG1, clone OCG4, 1:400, Thermo Fisher Scientific, Rockford, IL, USA), anti-osteopontin (OPN) (mouse monoclonal, clone 7C5H17, 1:200, Abcam, Inc., Cambridge, UK), anti-bone sialoprotein (BSP) (rabbit polyclonal, 1:50, Abcam, Inc., Cambridge, UK), anti-dentin sialophosphoprotein (DSPP) (rabbit polyclonal, 1:200, Abcam, Inc., Cambridge, UK), anti-type I collagen (Coll-1) (mouse monoclonal, clone (COL-1), 1:2000, Thermo Fisher Scientific, Rockford, IL, USA), anti-osterix (Osx) (rabbit monoclonal, 1:200, Abcam, Inc., Cambridge, UK), and anti-runt-related transcription factor 2 (Runx2) (rabbit monoclonal, 1:1000, Abcam, Inc., Cambridge, UK).

#### 4.4.2. Immunohistochemical Staining (IHC)

Immunohistochemical staining for bone-related proteins was performed on specimens before and after hDPSC osteogenic differentiation on PLCL and HYAFF-11 scaffolds and in a 2D cultured condition using the Universal Dako REAL EnVision Detection System. Peroxidase/DAB+, Rabbit/Mouse (Dako, Copenhagen, Denmark), and the following primary antibodies were used to detect: OCN, OPN, DSPP, Osx, Coll-I, BSP, and Runx2 proteins. hDPSC/PLCL and hDPSCs/HYAFF-11™ specimens were fixed in a 10% formalin solution (Merck, Saint Louis, MO, USA) for 15 min. The cytospin specimens were prepared from cells removed from a 2D culture and hDPSC/PLCL and hDPSC/HYAFF-11^TM^ constructs using a 0,25% trypsin-EDTA solution (Sigma-Aldrich, St. Louis, MO, USA). After cells were washed in PBS, the cytospin slides were prepared and fixed in a 10% formalin solution (Merck, Saint Louis, MO, USA) for 15 min. Next, endogenous peroxidase reactivity was blocked with the Dako REAL Peroxidase Blocking Solution (Dako, Copenhagen, Denmark); after that, the specimens were incubated with primary antibodies overnight at 4 °C. After washing with a 0.1 M Tris buffer, pH = 7.4 (TBS), the scaffold specimens and cell slides were incubated with Dako REAL EnVision/HRP, Rabbit/Mouse (Dako, Copenhagen, Denmark) for 30 min at RT. The antigen–antibody reaction was visualized using DAB (3,3 diaminobenzidine) (Dako, Copenhagen, Denmark) as a chromogen for four min at RT. The sections were counterstained with hematoxylin. The incubation buffer (TBS) without primary antibodies was used as a negative control [22].

#### 4.4.3. Immunohistochemical Staining Interpretation

The expression of the analyzed osteogenic proteins in the hDPSCs located on the membrane was assessed semiquantitatively, taking into account the staining intensity and the number of cells showing immunoreactivity for the analyzed osteogenic proteins: OCN, OPN, Osx, DSPP, Coll-I, BSP, and Runx2. The percentage of immunopositive cells was determined by counting the positive cells for the analyzed proteins versus the total number of cells visible in randomly selected areas of the hDPSC/scaffold constructs. In the cytospin slides, the percentage of positive cells for the analyzed proteins was determined by counting 1000 cells in a randomly selected field using an Olympus BX51 microscope (Olympus, Tokyo, Japan). Specimens with over 5% of cells showing positive immunostaining were considered positive. The intensity score was based on the color of the reaction, where no color = no immunostaining, light yellow color = weak (+), medium brown color = moderate (++), and brown color = strong (+++).

#### 4.4.4. Quantitative Polymerase Chain Reaction (qPCR) for Osteogenic Gene Expression

To compare the impact of two different biomaterials, PLCL and HYAFF-11™, on the osteogenic markers’ expression, hDPSCs were cultured for 21 days on biomaterials or in a plastic cell culture dish (control) in a complete hMSC osteogenesis induction medium. Total RNA was isolated and purified with the RNeasy Plus Mini Kit (Qiagen, Hilden, Germany), according to the manufacturer’s instructions. Then, a reverse transcription of 0.1 μg of total RNA was conducted with the use of the RevertAid First Strand cDNA Synthesis Kit (Thermo Fisher, Vilnius, Lithuania). The quantitative PCR for gene expression listed in Table 2 was performed on a ViiA 7 Real-Time PCR System (Applied Biosystems, Foster City, CA, USA) using Rotor-Gene SYBR Green (Qiagen, Hilden, Germany). The reactions were carried out three times with the following program running settings: 10 min of initial denaturation at 95 °C, followed by 40 cycles of 15 s denaturation at 95 °C; next, 1 min of annealing at 60 °C and then 40 s of extension at 72 °C. The levels of the housekeeping gene β-actin were used to normalize all qPCR product quantifications (∆CT), and the relative gene expression levels were calculated using the 2^−∆∆CT^ method.

### 4.5. Statistical Analysis

Data are presented as mean or median ± SD. The differences in the adhesion of hDPSCs on PLCL and on HYAFF-11^TM^ were determined using a t-Student test or a Kruskal–Wallis test. Statistical analysis was performed using Statistica TIBCO Software Inc., version 13 (Palo Alto, CA, USA). For the comparison of bone-related gene expressions, a one-way analysis of variance (one-way ANOVA) with Dunnet’s test for multiple comparison procedures was used. Statistical analysis was performed using GraphPad Prism version 7. A statistical significance was considered for *p*-values < 0.05.

## 5. Conclusions

Our study revealed that the structural variation of the hydrolytically modified nanofiber PLCL mats had a better impact on hDPSC adhesion compared to non-woven hyaluronan-based HYAFF-11™ meshes, as confirmed by SEM analysis. This phenomenon might be dependent on fiber size and the ability for cell–cell contact, providing a better environment for hDPSC proliferation and osteogenic differentiation, as confirmed by mRNA osteogenic gene expressions. These data demonstrate that the combination of PLCL or HYAFF-11™ with hDPSCs could be a promising strategy to generate bioimplants that favor cell adhesion, the upregulation of bone-related gene expression, and the activity of corresponding osteogenic proteins. However, as PLCL had a better impact on hDPSCs’ osteogenic potential than HYAFF-11™, the construct hDPSC/PLCL can be considered an innovative approach for bone defect repair.

## Figures and Tables

**Table 1:** Comparison of bone-related protein expression on hDPSCs grown on PLCL and HYAFF-11^TM^ scaffolds before and after osteogenic differentiation.

Immunoreactivity ( percentage of positive cells, mean +SD)															
Cultured condition	No. of donors	Runx2		Osx		Coll-I		BSP		DSPP		OPN		OCN	
		[%]	*p*	[%]	*p*	[%]	*p*	[%]	*p*	[%]	*p*	[%]	*p*	[%]	*p*
hDPSCs before differentiation	3	13.9 ± 5.46	0.0026 *	15.6 ± 7.26	0.0094 *	20.0 ± 7.07	0.0006*	18.3 ± 6.61	0.0061 *	18.9 ± 8.94	0.0006 *	13.3 ± 5.59	0.0041 *	23,3 ± 5.59	0.0158 *
hDPSCs on PLCL after differentiation	3	21.7 ± 6.32		44.4 ± 10.14		69.4 ± 6.67		63.3 ± 4.17		51.1 ± 9.28		50.6 ± 7.5		64.4 ± 6.82	
hDPSCs on HYAFF-11^TM^ after differentiation	3	21.1 ± 6.32	0.0115 ^	50.6 ± 7.26	0.0114 ^	54.4 ± 6.67	0.0037^	60.6 ± 4.17	0.0131 ^	53.3 ± 7.95	0.0154 ^	52.7 ± 7.5	0.0077 ^	63.9 ± 6.82	0.0027 ^
hDPSCs on PLCL after differentiation	3	21.7 ± 6.32	0.8422	44.4 ± 10.14	0.1774	69.4 ± 6.67	0.0002	63.3 ± 4.17	0.5889	51.1 ± 9.28	0.6317	50.6 ± 7.5	0.7556	64.4 ± 6.82	0.9291
hDPSCs on HYAFF-11^TM^ after differentiation	3	21.1 ± 6.32		50.6 ± 7.26		54.4 ± 6.67		60.6 ± 4.17		53.3 ± 7.95		52.7 ± 7.5		63.9 ± 6.82	

[%]—mean ± SD of positive hDPSCs; *p* < 0.05 statistically significant differences; *p*–t-Student test; * comparison between hDPSCs before differentiation and hDPSCs on PLCL after differentiation; ^ comparison between hDPSCs before differentiation and hDPSCs on HYAFF-11^TM^ after differentiation.

**Table 2:** Primers used in qPCR.

Gene	Primer Sequences (5′-3′)	Amplicon Length (bp)
*β-actin*	F:5′-AGGGCAGTGATCTCCTTCTGCATCCT-3′ R:5′-CCACACTGTGCCCATCTACGAGGGGT-3′	1852
*Runt-related transcription factor 2*	F:5′-GTGGACGAGGCAAGAGTTTCA-3′ R:5′-CCGTGTCTGTCTTCGAACTAC-3′	187
*Collagen type I*	F:5′-AGGTGCTGATGGCTCTCCT-3′ R:5′-TGTTCCCACTTTCACCAGG-3′	178
*Osterix*	F:5′-ATCCAGCCCCCTTTACAAGC-3′ R:5′-TAGCATAGCCTGAGGTGGGT-3′	408
*Osteocalcin*	F:5′-GCAGGTGCGAAGCCCAGCGGTGCAGAG-3′ R:5′-GGGCTGGGAGGTCAGGGCAAGGGCAAG-3′	562
*Osteopontin*	F:5′-ATCACCTGTGCCATACCA-3′ R:5′-CATCTTCATCATCCATATCATCCA-3′	1823
*Bone sialoprotein*	F:5′-TCACTGGAGCCAATGCAGAA-3′ R:5′-TGGAGAGGTTGTTGTCTTCGAG-3′	1573
*Dentin sialophosphoprotein*	F:5′-GGCAGTGCATCAAAAGGAGC-3′ R:5′-TGCTGTCACTGTCACTGCTG-3′	4331

## Data Availability

All data generated or analyzed during this study are included in this article.
